# Use of noninvasive positive pressure ventilation during pregnancy: Case series

**DOI:** 10.4103/1817-1737.30358

**Published:** 2007

**Authors:** Mariam A. Al-Ansari, Akmal A. Hameed, Suhaila E. Al-jawder, Hassan M. Saeed

**Affiliations:** Salmaniya Medical Complex, Ministry of Health, Kingdom of Bahrain

**Keywords:** Acute respiratory failure, noninvasive ventilation, outcome, pregnancy

## Abstract

Mechanical ventilation is commonly required in critically ill pregnant patients, requiring ICU admission, with higher morbidity and mortality related to airway management. Alternatively, noninvasive positive pressure ventilation (NIPPV) is increasingly used to treat nonpregnant patients. Pregnancy has been a contraindication to its use. We would like to report a case series of successful use of NIPPV in pregnancy.

NIPPV is increasingly used to treat hypoxemic respiratory failure. It has rarely been used during pregnancy. On the other hand, acute respiratory failure (ARF) remains a leading cause of ICU admission in obstetric patients. The use of NIPPV in managing ARF in pregnant patients was not investigated. We report the outcome of treatment with NIPPV of four sickle cell disease pregnant patients with ARF caused by acute chest syndrome. Median APACHE II score for the four cases was 27. Intubation was avoided in all cases. None had aspiration. Mean duration of NIPPV was 40 h with ICU discharge after a mean of 4 days.

Acute respiratory failure (ARF) occurs more frequently in pregnant than nonpregnant patients, requiring admission to intensive care unit (ICU), with higher morbidity and mortality.[1,2] Failed intubation in this population carries mortality risk eight times higher than in nonpregnant patients.[3] Several factors have been identified that may contribute to the higher morbidity and mortality associated with airway management in the pregnant patient.[4]

To the best of our knowledge, noninvasive positive pressure ventilation (NIPPV) has only been used during pregnancy in non-ICU setting.[5,6] It has been increasingly used to treat ARF in a wide variety of conditions with better outcome compared to invasive ventilation.[7] *Some have* suggested, based on experience, that moderate cases of acute chest syndrome (ACS), 81% of whom will require mechanical ventilation, may respond to noninvasive positive pressure ventilation.[8] Because of the frequency of SCD in our area and the frequent admission of pregnant SCD patients with ACS to our ICU, we decided to look at the use of NIPPV in these patients. Hospital research committee approved our proposal and waived the requirement of consent. In this case series, we report the initial four cases of pregnant patients with ACS causing ARF who were successfully and safely managed by NIPPV in the ICU.

## Patients

Four consecutive pregnant patients with ACS and ARF admitted to the ICU from February 2003 until May 2004 were included. All patients were young adults with only medical history of sickle cell disease. None had history suggestive of obstructive sleep apnea. ARF was defined by severe hypoxemia (i.e., PaO_2_/ fraction of inspired oxygen [FiO_2_] ratio less than 200) with respiratory distress (respiratory rate 35 breaths/min or more). The diagnosis of ACS was based on the presence of a *new* infiltrate on chest X-ray associated with one or more of new symptoms like fever, cough, sputum production, dyspnea or hypoxia.[9] NIPPV was the first line mechanical ventilatory support to be offered if FiO_2_ supplement through non-rebreather face mask failed to maintain oxygen saturation above 92% on the monitor in an awake and cooperative patient.

## Treatment

A uniform standardized treatment protocol was applied for all patients. Symptomatic treatment included IV hydration with hypotonic fluids to achieve euvolemic state, increasing oxygen administration to keep O_2_ saturation above 95% on the monitor, patient-controlled analgesia and folic acid. Antibiotic (a combination of a macrolide and a betalactam) was started immediately after blood and urine samples were taken for culture. All patients received early exchange transfusion, before getting initial Hb S level upon admission. Target was to keep Hb S below 30%. Deep venous thrombosis prophylaxis was started in all patients. All included patients were kept on NIPPV through face mask using CPAP with pressure support to keep tidal volume at around 300–350 ml and a respiratory rate below 35/min with an oxygen saturation above 92% throughout the NIPPV trial. None of the patients had an ECHO done as the clinical impression was of ACS and none had history suggestive of other diagnostic possibility. All patients were closely followed up by an obstetrician on regular basis. Indication for intubation was a poor ABG in the first hour after the start of NIPPV. NIPPV was weaned off when the respiratory rate and oxygen requirement decreased, along with the achievement of ability to maintain oxygen saturation above 95% on maximum oxygen requirement of 8 liters/min through face mask no less than 6–8 h after the start of NIPPV.

## Results

Table 1 shows the base line characteristics of the four treated cases. In all cases, NIPPV had to be instituted within 2–3 h of ICU admission. During this time, the standardized treatment protocol was applied. There were no problems with mask fit or maintenance of the positive pressure. None of the patients subsequently had positive blood cultures. Summary of the outcome of NIPPV use in the four treated patients is shown in Table 2.

**Table 1:** Base line characteristics of the four cases treated using noninvasive positive pressure ventilation

Patient Characteristics	Case 1	Case 2	Case 3	Case 4
Age (years)	32	39	29	26
Age (years)	32	39	29	26
Reason for hospital admission	Fever + VOC	Dyspnea	VOC SOB	SOB, Palpitation
Reason for ICU admission	Pneumonia Respiratory distress	ACS	ACS	Hypoxia
APACHE II	29	27	29	24
Pregnancy (weeks)	28	32	29	32
Presence of previous	1	1	2	1
ACS attack				
Was patient on hydroxyurea prior to hospital admission	Y	N	Y	N
Laboratory values on hospital admission: Hb level (g/dL)	9.9	10.6	11.3	9.6
Hct level(%)	39	33	42	31
WBC counts (× 10^−6^/Liter)	12.9	16.3	11.2	4.3
Platelets count (× 10^−6^/Liter)	150	78	126	66
LDH, IU/L	663	437	332	351
Hermoglobin S level on	36	43	31	52
ICU admission (%)			
pO2/FiO2 ratio prior to NIPPV	195	176	102	198

SOB: Shortness of breath, VOC: Vasocclusive crisis, ACS: Acute chest syndrome, Y: Yes, N: No, LDH: Lactic dehydrogenase, NIPPV - NonInvasive positive pressure ventilation

**Table 2:** Outcome of patients treated using noninvasive positive pressure ventilation

Outcome measure	Case 1	Case 2	Case 3	Case 4
pO2/FiO2 ratio within	222	234	189	246
first hour of NIPPV			
pO2/FiO2 ratio within	298	288	226	293
6 hour of NIPPV			
Duration of NIPPV	49	44	30	41
Use (hours)			
Use of dopamine	Y	N	Y	N
Duration of use of	2	-	4.5	-
inotrops (hours)			
Need to shift from NIIPV	N	N	N	N
to invasiveventilation			
Development of aspiration	N	N	N	N
Duration of ICU stay (days)	5	5	3	4
Duration of Hospital stay (days)	14	11	9	9

Y: Yes, N: No, SVD: Spontaneous vaginal delivery, LSCS: Lower segment cesarean section, NIPPV - Noninvasive positive pressure ventilation

Retrospective review of three comparable cases, managed in our unit with invasive positive pressure ventilation prior to this trial of NIPPV use, revealed a potential outcome benefit with the use of NIPPV [Table 3].

**Table 3:** Comparison of outcome between the NIPPV group and comparable previously treated patients using invasive ventilation

Outcome measure	NIPPV group	Invasive group
Mean APACHE score	27	25
Mean duration of ventilator support (hours)	40	36
Mean duration of ICU stay (days)	4	7
Mean duration of hospital stay (days)	10	14

NIPPV - Noninvasive positive pressure ventilation

## Discussion

NIPPV has had limited application in pregnant patients due to the perceived risk of aspiration. The current case series has its own limitations in providing definitive recommendations for the indications for and use of NIPPV in the pregnant patient with ARF. A follow-up well-conducted study would be of great use in determining when and how to use this management approach in pregnant patients with sickle cell disease presenting with ACS.

Obstetric patients with preexisting medical problems are more likely to require intensive-care support than those without preexisting medical conditions.[10] Sickle cell disease is the most common major underlying chronic medical condition in obstetric patients admitted to our ICU. ACS is believed to be a specific form of acute lung injury that can progress to acute respiratory distress syndrome causing ARF.[11] Because there are no absolute contraindications to the use of NIPPV and the boundaries for its use continue to expand, we decided to look at the use of NIPPV in pregnant patients with ARF caused by ACS.

Pregnant patients are at risk of aspiration. It is a serious complication of pregnancy and is a frequent cause of indirect obstetric death. All the patients in this series were kept nil per mouth and treated with placement of distal gastric tubes for decompression. None developed aspiration pneumonia.

Young adults have a lower incidence of ACS, but it tends to be more severe and is often fatal.[12] Pregnant patients with ACS causing ARF admitted to ICU need aggressive and timely intervention from a multidisciplinary team of physicians to minimize the morbidity and mortality associated with this devastating complication of sickle cell disease. Moreover, acute respiratory failure contributes substantially to maternal morbidity and mortality; it can also harm the fetus by compromising fetal oxygen delivery. Upon follow-up, only one patient had LSCS. Both patients and the babies were alive and healthy at 6 months after delivery.

A significant incidence of right ventricular dysfunction and pulmonary hypertension in asymptomatic patients with sickle cell anemia has been reported.[13] In the setting of severe, acute lung disease, this quiescent pulmonary hypertension can cause right ventricular dysfunction that is severe enough to cause circulatory compromise. Hypotension is therefore possible. Two of our patients required short course of inotropic support. However, none of the four deteriorated to the extent of requiring invasive mechanical ventilation.

Critical illness in pregnant women poses special challenges. Physiologic changes that affect cardiovascular and respiratory function are normal during pregnancy. Through interaction with preexisting or new co-morbidities, those changes can give rise to life-threatening complications. Always present, too, is the need to consider the effects of both disease and its treatment on fetal development and outcome. In this small case series, the use of NIPPV was found to be safe and successful with good maternal and fetal outcome in pregnant patients with ARF caused by ACS.

Currently there is not enough evidence to support safe use of NIPPV in ARF in a pregnant patient. The current case series provides the best available evidence to support the use of NIPPV in ARF during pregnancy. In closely monitored pregnant patients with ARF, NIPPV seems to have the potential to shorten ICU and hospital stay. A well-conducted randomized controlled clinical trial is required to confirm this finding.
